# Experiences of International and Puerto Rican Medical Graduates in the United States: A Cross-Sectional Survey

**DOI:** 10.1007/s11606-026-10392-9

**Published:** 2026-04-14

**Authors:** Coral Olazagasti, Claudia Villa Celi, Arun Mahtani, Lauren Kiel, Ana I. Velazquez, Miki Horiguchi, Arthi Sridhar, Mariana Gonzalez, Carolina Bernabe, Oyepeju Abioye, Nazli Dizman, Narjust Florez

**Affiliations:** 1 Sylvester Comprehensive Cancer Center, University of Miami Miller School of Medicine, Miami, FL USA; 2 Cleveland Clinic Florida, Weston, FL USA; 3 New York University, New York, NY USA; 4 University of Colorado Anschutz Medical Campus, Aurora, CO USA; 5 University of California San Francisco, San Francisco, CA USA; 6 UT Southwestern Medical Center, Dallas, TX USA; 7 Universidad Americana, Managua, Nicaragua; 8 Montefiore Einstein Cancer Center, Bronx, NY USA; 9 Allegheny Health Network, Pittsburgh, PA USA; 10 Univeristy of Texas MD Anderson Cancer Center, Houston, TX USA; 11 Harvard Medical School, Boston, MA USA

## Abstract

**Purpose:**

International medical graduates (IMGs) and Puerto Rican medical graduates (PRMGs) are integral to the United States (U.S.) physician workforce yet face unique immigration-related and professional challenges. We conducted a study to investigate reasons for migration, perceived barriers, discrimination, satisfaction, and factors influencing IMGs’ and PRMGs’ decisions to remain in the U.S. versus return to their home countries.

**Methods:**

We conducted a cross-sectional, online survey of foreign-born physicians who obtained their medical degrees outside the continental United States and were currently training or practicing in the U.S. The survey captured demographic and professional characteristics, migration history, cultural adaptation, discrimination, and overall professional experiences. Descriptive statistics summarized responses. Among physicians who completed training and were practicing independently in the U.S., we compared overall experience ratings and discrimination-related distress between the training and independent practice phases using the Wilcoxon signed-rank test and McNemar’s test.

**Results:**

Of 352 respondents, most were IMGs (82.1%) and 16.8% were PRMGs. Nearly half reported racial and/or ethnic discrimination (49.6%) and language discrimination (37.8%) during training. In paired analyses among those currently in independent practice, overall experience ratings shifted from the training to independent practice phase, with “excellent” ratings more common during training than independent practice. Among participants who experienced language discrimination in both phases, distress levels decreased over time. Despite high rates of discrimination and pressure to assimilate, most participants reported excellent (63.6%) or good (52.9%) overall experiences during training and practice, respectively. Only 9.3% had returned to their home country, most of whom reported extreme happiness after returning.

**Conclusion:**

High rates of racial, ethnic, gender, and language discrimination, along with pressures to assimilate, characterize IMGs’ and PRMGs’ journeys in the U.S. healthcare system. Nevertheless, most report high professional satisfaction. Interventions are needed to address discrimination, support cultural identity, and promote sustainable careers for this essential segment of the U.S. workforce.

**Supplementary Information:**

The online version contains supplementary material available at 10.1007/s11606-026-10392-9.

## INTRODUCTION

As of 2021, approximately 28% of the 949,546 practicing physicians in the United States (U.S.) are foreign trained^1,2^. Physicians who earn their medical degrees outside of the U.S. and Canada are known as International Medical Graduates (IMGs)^3–5^, while those graduating from Puerto Rico are referred to as Puerto Rican Medical Graduates (PRMGs).

Economic and political instabilitystability, better professional opportunities, and poor training and work environments in their home countries are among the reasons IMGs migrate to the U.S.^6–10^ PRMGs similarly cite factors such as job security, safety from violence, high cost of living, and low physician pay in Puerto Rico^11^. However, despite escaping challenging native circumstances, IMGs and PRMGs often face barriers associated with immigration and encounter vastly different professional experiences compared to graduates from the continental U.S. These barriers include discrimination during residency selection and training, stress, burnout, lower-quality training placements, and assignment to less competitive specialties^12–15^. Even after securing residency positions, IMGs and PRMGs frequently experience bias, financial challenges, suboptimal mentorship, and career advancement difficulties, as well as continued discrimination from colleagues and patients throughout their careers^12–22^.

These challenges take a toll on IMGs’ emotional well-being, contributing to a high risk for burnout. Due to the persistence of these stressors throughout their independent careers, many IMGs experience emotional distress as they navigate the U.S. graduate medical education (GME) system^20,23,24^. Data regarding burnout in IMGs remains understudied and can be confounded by selection bias, due to fear of retaliation. Though scarce, literature comparing the rates of career satisfaction of IMG physicians with their USMG counterparts exists. One study revealed that IMGs had significantly lower rates of career satisfaction than USMGs, even after adjusting for physician characteristics and practice environment^20^. Another study found that despite reporting a higher likelihood of having adequate time to spend with patients (73% versus 66%; %, *p* = 0.0041) and a greater ability of forming relationships with their patients (83% versus 78%, *p* = 0.0096) than USMGs, IMGs were significantly more dissatisfied with their overall medical careers than USMGs (20% versus 16%, *p*= 0.0190)^25^. Moreover, circumstances for IMGs are potentially deteriorating. On January 26th, 2022, the National Board of Medical Examiners changed the scoring system of the U.S. medical licensing examination (USMLE) from numerical scores to pass/fail, thereby disproportionately impacting IMGs and PRMGs by limiting their ability to stand out during residency and fellowship applications^26,27^. As up to 78% of programs considered this score to rank applicants, these scores often helped ensure IMGs and PRMGs a residency/fellowship position. Combined with high examination and visa processing fees, these hurdles only exacerbate the difficulties faced by these physicians^19^.

IMGs and PRMGs play a critical role in the U.S. healthcare system, particularly in underserved areas. They are more likely than U.S. medical graduates to serve low-income communities and treat non-White, non-English-speaking, and uninsured populations^5,28–31^. IMGs and PRMGs also comprise significant portions of specialties like geriatric medicine (51.3%), nephrology (51.0%), endocrinology (42.2%), and rheumatology (35.3%)^32^. Additionally, many enter primary care, with 39.8% practicing internal medicine and 24.2% in family medicine^28,33^, often through the U.S. Conrad 30 Waiver Program^4^. This improves access to care in underserved and rural areas, addresses primary care shortages, and reduces barriers to culturally competent care^34–36^. As approximately 83 million Americans lack access to primary care physicians due to their geographic location, the contribution of IMGs and PRMGs in these areas is of such paramount significance that, without IMGs, the percentage of physician shortage would increase to almost 45%^28,29,37^. Having a diverse workforce has been shown to improve patient outcomes, satisfaction, and treatment adherence, helping to reduce existing racial and ethnic disparities in healthcare^38^. Without their contributions, the U.S. physician shortage would increase significantly, leaving millions without adequate access to care and widening existing healthcare disparities. As the U.S. Census Bureau projects that 56% of the US population will be of a racial minority and 19% will be foreign-born by the year 2060^31,39^, it is increasingly important to ensure that the presence of racially and language-concordant physicians, associated with improved patient outcomes and satisfaction, is amplified in the workforce^16^.

Recognizing and addressing the unique needs of these physicians are critical to fostering a diverse and equitable healthcare workforce. Therefore, we sought to explore the migration journeys, challenges, and professional experiences of IMGs and PRMGs across the U.S. healthcare system.

## METHODS

This was a cross-sectional study designed to comprehensively investigate the reasons behind migration, challenges, satisfaction, and the factors influencing the decision of IMGs and PRMGs to remain in the U.S. versus returning to their home countries. The study was approved with a waiver of documentation of informed consent from the Institutional Review Board of the University of Miami.

The survey was distributed using the premium SurveyMonkey® platform. Eligibility criteria included adults who were born and obtained their medical degrees outside the continental U.S. but were either undergoing or had completed residency and/or fellowship training or practicing independently as attending physicians in the U.S. Participants were required to be proficient in English to complete the survey. Invitations were extended to individuals in both academic and community practice settings.

To identify IMG-friendly residency and fellowship programs, we utilized the Fellowship and Residency Electronic Interactive Database™ (FREIDA), which provides detailed information on over 13,000 ACGME-accredited residency and fellowship programs across the U.S. Using this database, we compiled a list of IMG-friendly programs along with the contact information of program directors and coordinators. Weekly emails containing the study logo, a cover letter, and the survey link were sent to these program directors and coordinators, requesting them to share the survey with their residents and fellows. Additionally, each study investigator contributed to a distribution list of IMGs and PRMGs by including known colleagues and peers. Personalized email invitations containing the survey link were sent weekly to individuals on this list. As part of broader outreach efforts, messaging apps like WhatsApp© were used to share the survey with IMG and PRMG groups, and promotional materials were disseminated via social media platforms such as X (formerly Twitter), Facebook, Instagram, and Reddit.

To prevent ineligible responses, the survey link was not shared publicly. Instead, interested participants were required to contact the study team via email or direct message to request access. A member of the study team screened every participant for eligibility before sharing the survey link. The survey remained open from September 4 to December 4, 2023. Regular data quality reviews were conducted during this period to ensure the legitimacy of responses.

The survey comprised 51 multiple-choice and open-ended questions encompassing demographic information, professional status, migration timelines, initial U.S. migration locations, motivations for migration (e.g., lack of opportunities, financial growth, fear of delinquency or civil war), cultural adaptation (e.g., cuisine, dress code, traditions), experiences of discrimination during training (e.g., gender, racial/ethnic, and language barriers), and overall professional experiences in the U.S. (Appendix 1). To accommodate the sensitive nature of some questions related to negative institutional experiences, participants were allowed to skip questions.

Descriptive statistics were used to summarize participant demographic characteristics and survey responses. Overall experience ratings during the residency and/or fellowship training phase and the independent practice phase were compared using the Wilcoxon signed-rank test to assess within-individual changes in ordinal responses. For each type of discrimination, McNemar’s test was used to evaluate whether the proportion of participants who experienced it differed between the two phases. Among those who reported experiencing discrimination in either phase, distress ratings were compared using the Wilcoxon signed-rank test. Missing data were excluded from the analysis, and percentages reported reflect the number of respondents who had chosen to answer each specific question. All analyses were conducted using R version 4.2.3 (R Foundation for Statistical Computing, Vienna, Austria). A two-sided *p*-value of < 0.05 was considered statistically significant.

### Country of Origin and Subgroup Analyses

Participants reported their country of birth, which we grouped into predefined world regions: Latin America and the Caribbean (e.g., Antigua, Argentina, Brazil, Chile, Colombia, Cuba, Dominican Republic, Ecuador, El Salvador, Guatemala, Honduras, Jamaica, Mexico, Nicaragua, Panama, Peru, Trinidad and Tobago, Uruguay, Venezuela, U.S. Puerto Rico, and U.S. Virgin Islands), South Asia (Bangladesh, India, Nepal, Pakistan, Sri Lanka, Kashmir, and Myanmar), Middle East/North Africa (Egypt, Iraq, Israel, Jordan, Kuwait, Lebanon, Saudi Arabia, Sudan, Turkey, and the United Arab Emirates), Sub-Saharan Africa (Cameroon, Ethiopia, Ghana, Kenya, and Nigeria), East and Southeast Asia (China, Japan, and Taiwan), Europe and Eurasia (Armenia, Azerbaijan, France, Germany, Greece, Hungary, Ireland, Italy, Kazakhstan, Moldova, Romania, Russia, Ukraine, the United Kingdom, and responses coded as “Europe”), North America (United States and Canada), and unknown/missing. We summarized the distribution of country of birth by region and, in exploratory analyses, descriptively examined selected outcomes stratified by PRMG versus other IMGs; these results are presented in the supplementary material.

## RESULTS

### Baseline Demographics

A total of 362 participants began the survey. Of those, 352 completed 100% of the survey and will be included in this analysis.

As depicted in Table 1, most participants were 25–34 years old. Our sample was equitable and diverse; nearly half of participants identified as women, and most self-identified as White race, and more than half self-identified as Hispanic or Latino ethnicity. Participants were predominantly attending physicians practicing in an academic setting.

**Table 1:** Participant Demographics

**Variable**	**Category**	**Total** *N* = 352 (%)
Age	25–34	155 (44.0)
	35–44	142 (40.3)
	45–54	29 (8.2)
	55–64	14 (4.0)
	65 +	12 (3.4)
Gender	Man	177 (50.3)
	Woman	175 (49.7)
Race	White	158 (46.2)
	Black or African American	21 (6.1)
	American Indian or Alaska Native	3 (0.9)
	Hispanic or Latino	16 (4.7)
	Middle Eastern or North African	10 (2.9)
	Asian or Asian American	82 (24.0)
	Multiracial	4 (1.2)
	None of the above	48 (14.0)
Ethnicity	Hispanic or Latino	180 (51.7)
	Non-Hispanic White	53 (15.2)
	Non-Hispanic Black	20 (5.7)
	Non-Hispanic Other	83 (23.9)
	None of the above	12 (3.4)
Region of birth	Latin America and the Caribbean	124 (35.2)
	North America	71 (20.2)
	South Asia	61 (17.3)
	Middle East and North Africa (MENA)	35 (9.9)
	Europe and Eurasia	20 (5.7)
	Sub-Saharan Africa	11 (3.1)
	East and Southeast Asia	3 (0.9)
	Unknown	27 (7.7)
International medical graduate	No	63 (17.9)
	Yes	289 (82.1)
Puerto Rican medical graduate	No	289 (82.6)
	Yes	63 (17.9)
Title	Attending Physician	180 (51.1)
	Fellow Physician	49 (13.9)
	Resident Physician	123 (34.9)
Years in independent practice	Less than 1 or 1	54 (15.3)
	2–5	91 (25.9)
	6–10	31 (8.8)
	More than 10	54 (15.3)
	Currently in a training program	115 (32.7)
	None of the above	7 (2.0)
Current practice setting	Academic setting	188 (53.9)
	Community or Private practice	67 (19.2)
	Hybrid or academic affiliate	23 (6.6)
	Independent practice	27 (7.7)
	Public or Federally Qualified Health Center	11 (3.2)
	VA	4 (1.1)
	None of the above	29 (8.3)

Countries of birth spanned all major world regions (Appendix Table 2). Nearly half of participants were born in Latin America and the Caribbean (171/352, 48.6%), followed by South Asia (61/352, 17.3%) and the Middle East/North Africa region (35/352, 9.9%). Smaller proportions were born in Europe and Eurasia (20/352, 5.7%), Sub-Saharan Africa (11/352, 3.1%), East and Southeast Asia (3/352, 0.9%), and North America (Canada or the continental United States; 24/352, 6.8%), while 27 respondents (7.7%) did not specify their country of birth. As expected, given our inclusion criteria, 82.1% of respondents were IMGs and 16.8% were PRMGs.

For the whole cohort, the most common reasons for migration to the U.S. were seeking better medical education, seeking professional gains, and a lack of opportunities in participants’ home countries (Table 2). As seen on Appendix Table 2, most participants from Europe and Asia (45%) selected family reasons as the most common reason to emigrate to the U.S.

**Table 2:** Details About the Migration

	*N*(%)
Reason for the migration*, *n* = 352	
Health reasons	1 (0.3)
Seeking refugee	4 (1.1)
Leaving gang wars	11 (3.1)
Avoiding civil wars	16 (4.5)
Escaping political oppression	39 (11.1)
Looking for opportunities for family	64 (18.2)
Family reasons	72 (20.5)
Wanting professional recognition	72 (20.5)
Pursuing financial gains	73 (20.7)
Lack of opportunities in home country	152 (43.2)
Seeking professional gains	193 (54.8)
Looking for better education	208 (59.1)
Timing of the migration,*n* = 352	
Elementary school	2 (0.6)
High school	3 (0.9)
College	6 (1.9)
After completing medical school	247 (70.2)
After completing residency	52 (14.8)
After completing fellowship	21 (6.0)
None of the above	21 (6.0)

*Multiple choice question

Upon migration, many respondents reported having to modify aspects of their communication style, such as avoiding idioms, adjusting their accent, or changing their tone of voice, to align with perceived U.S. professional norms. A substantial proportion also described altering their dress, adapting to new communities and traditions, and celebrating U.S. holidays. Although a minority reported needing to learn or significantly improve their English after migrating, this was frequently associated with slight to moderate stress (Table 2).

### Overall Experiences

Training and independent practice status were available for 305 participants. Of these, 121 (39.7%) had completed U.S. training and were practicing independently at the time of the survey. Among this group, most rated their training experience as excellent, whereas independent practice was more commonly rated as good (Table 3).

**Table 3:** Decision Factor of Going Back Home Country and Staying in the U.S

Variable	*N* (%)
Decision factor of going back home country*, *n* = 352	
Toxic work environment	12 (3.4)
Family support	14 (4.0)
Language	28 (8.0)
Leadership opportunity	28 (8.0)
Partners preference	29 (8.2)
Visa/immigration status	38 (10.8)
Professional opportunity	42 (11.9)
Cost of living	52 (14.8)
Family illness	55 (15.6)
Food	61 (17.3)
Desire to raise children in home country	74 (21.0)
Feelings of isolation/lack of community	76 (21.6)
Quality of life	93 (26.4)
Culture	105 (29.8)
Family	237 (67.3)
Decision factor of staying in the U.S.*, *n* = 352	
Refugee status	7 (2.0)
Looking for a change	23 (6.5)
Avoiding political situations	53 (15.1)
Partner is not willing to move back home	55 (15.6)
Safety from wars violence	57 (16.2.)
Prefer to raise my family here	69 (19.6)
Lack of training opportunities in home country	87 (24.7)
Inability to pursue the same professional career	73 (20.7)
Professional recognition	102 (29.0)
Greater pay at work	126 (35.8)
Ability to seek professional growth promotion opportunities	129 (36.6)
Financial gain	144 (40.9)
Better and more work opportunities	156 (44.3)

*Multiple choice question

Within-individual comparisons revealed a significant decline in overall experience ratings from training to independent practice (*p* < 0.001). For example, the participants’ rating their experience as excellent decreased from 63.6% during training to 38.0% during independent practice, while the participants’ rating their experience as good increased from 27.3% to 52.9%.

### Distress and Discrimination

Discrimination during training was widespread. Among participants who completed both phases in the U.S. (*n* = 121), nearly half (49.6%) reported racial or ethnic discrimination during training, typically accompanied by moderate distress (Table 3). More than one quarter (28.8%) experienced gender discrimination—reported by both men and women—and over one third (37.8%) experienced language discrimination, most often related to accent.

These patterns continued into independent practice. Reported racial/ethnic discrimination remained common (47.8%), and gender discrimination persisted at similar levels (28.0%). Language discrimination decreased slightly (31.5%), and notably, distress associated with language discrimination significantly declined from training to practice (*p*=0.023), shifting from predominantly moderate distress to predominantly mild distress.

### Personal and Professional Satisfaction

**Figure 1:**
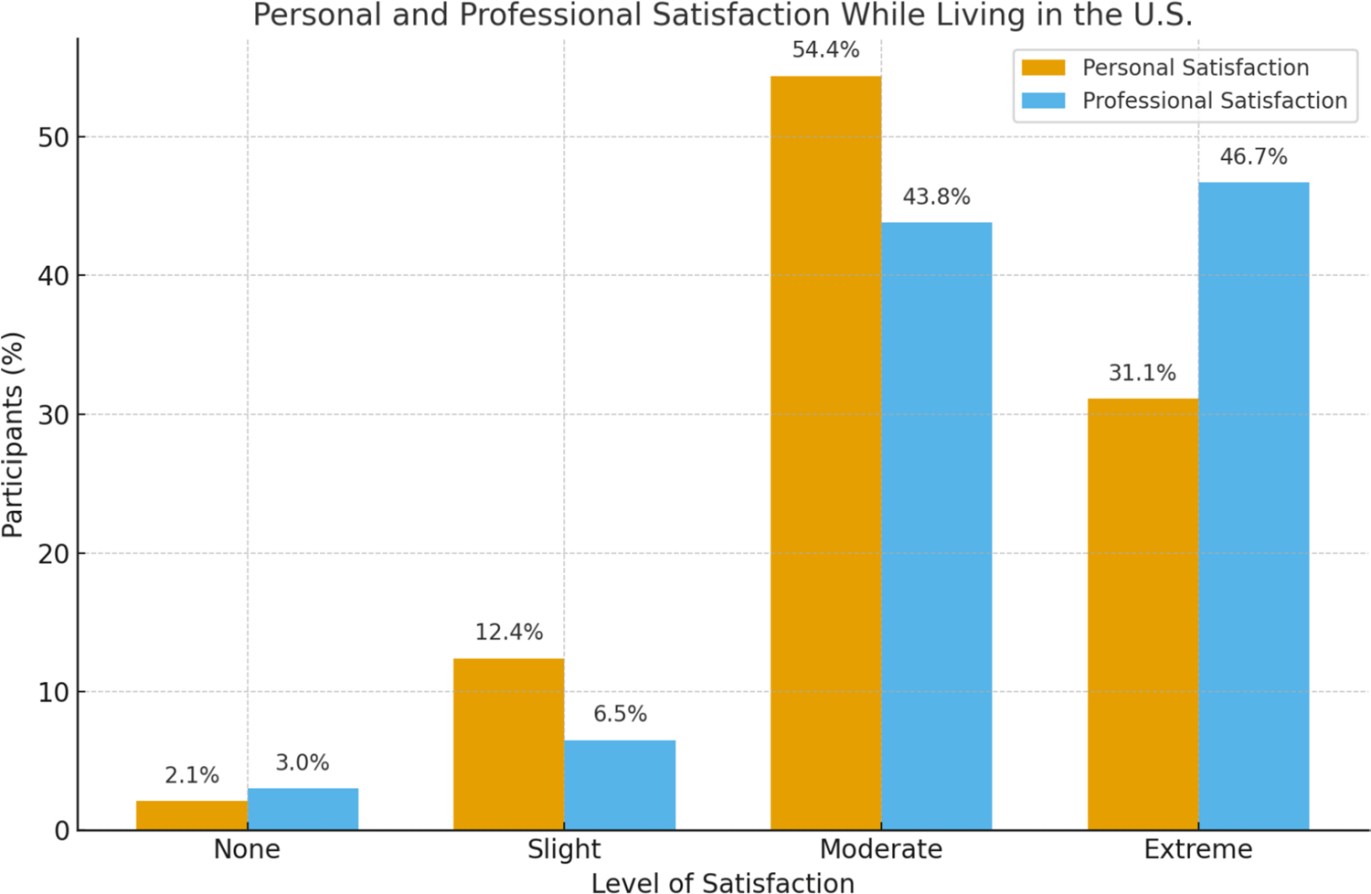
Personal and professional satisfaction while living in the United States. Bar chart displaying the distribution of participants’ self-reported personal and professional satisfaction while living in the U.S. The *x*-axis represents the level of satisfaction (“None,” “Slight,” “Moderate,” and “Extreme”), and the *y*-axis represents the percentage of participants. Personal and professional satisfaction are shown separately, with a legend included to distinguish between the two. Error bars are not shown, as values represent categorical responses.

Despite experiencing discrimination, participants expressed high levels of satisfaction with life and work in the U.S. Most reported moderate personal satisfaction, whereas professional satisfaction was substantially higher, with over 90% reporting moderate or extreme professional satisfaction (Fig. 1).

### Factors Influencing Staying Versus Returning Home

Family considerations overwhelmingly shaped participants’ desire to return home; 67.3% endorsed family as a major motivator (Table 4). Other factors included culture, quality of life, and feelings of isolation or lack of community support in the U.S. Appendix Table 3 from the supplementary analysis depicts the factors influencing staying versus returning home by region.

**Table 4:** Experience in the Residency and/or Fellowship Training and Independent Practice in the U.S. Among Those Who Completed Training and Currently Practicing Medicine Independently in the U.S. (*n* = 121)

Variable	Category	Training phase *N* (%)	Independent practice phase *N* (%)	*p*-value*
Overall experience in US	Poor	0	2 (1.7)	** < 0.001**
	Average	11 (9.1)	9 (7.4)	
	Good	33 (27.3)	**64 (52.9)**	
	Excellent	**77 (63.6)**	46 (38.0)	
Racial/ethnic discrimination**	Did not experience	58 (50.4)	60 (52.2)	0.838
	Experienced	57 (49.6)	55 (47.8)	
Distressed caused by racial/ethnic discrimination***	No distress	2 (4.5)	1 (2.3)	0.790
	Mild distress	24 (54.5)	25 (56.8)	
	Moderate distress	15 (34.1)	15 (34.1)	
	Severe distress	3 (6.8)	3 (6.8)	
Gender discrimination**	Did not experience	84 (71.2)	85 (72.0)	1.000
	Experienced	34 (28.8)	33 (28.0)	
Distressed caused by gender discrimination***	No distress	1 (4.3)	1 (4.3)	0.790
	Mild distress	6 (26.1)	8 (34.8)	
	Moderate distress	12 (52.2)	9 (39.1)	
	Severe distress	4 (17.4)	5 (21.7)	
Language discrimination**	Did not experience	69 (62.2)	76 (68.5)	0.211
	Experienced	42 (37.8)	35 (31.5)	
Distressed caused by language discrimination***	No distress	1 (3.7)	1 (3.7)	**0.023**
	Mild distress	9 (33.3)	**16 (59.3)**	
	Moderate distress	**14 (51.9)**	7 (25.9)	
	Severe distress	3 (11.1)	3 (11.1)	

Footnotes:

*Wilcoxon signed-rank test and McNemar’s test were used, as appropriate

**The denominators for the reported proportions include only participants who responded to the relevant questions in both phases, as required for paired comparisons

***The denominator includes only those who reported experiencing discrimination in both the training and independent practice phases, as required for paired comparisons

Motivations to remain in the U.S. were largely professional. High proportions cited better work opportunities (44.3%), financial gain (40.9%), opportunities for professional advancement (36.6%), and greater pay (35.8%) as reasons to stay.

Only a minority had already returned to their home country, and about 10% were actively considering returning. More than one quarter (28.7%) intended to return someday, though without a defined timeline, while 52.2% reported no plans to return. Among those who had returned (*n* = 32), over 90% described themselves as extremely or moderately happy with their decision, with very few expressing regrets.

## DISCUSSION

This national cross-sectional study provides one of the most comprehensive examinations to date of the migration journeys, professional challenges, and lived experiences of IMG and PRMG physicians across the U.S. healthcare system. We found that discrimination, particularly racial/ethnic, gender-based, and language-related, was common during both training and independent practice and that pressures to assimilate to perceived U.S. norms were widespread. Despite these challenges, most participants reported favorable overall experiences during training and high levels of professional satisfaction in independent practice, although personal satisfaction lagged behind. These findings highlight the complex and often paradoxical experiences of foreign-born physicians, who simultaneously navigate professional growth and systemic barriers.

A key contribution of this study is the demonstration that discrimination persists beyond training and into independent practice, where nearly half of participants continued to report racial/ethnic discrimination and more than a quarter reported gender discrimination. These experiences mirror prior literature documenting bias against IMGs during residency selection and clinical training, including discriminatory attitudes among program directors, biased ranking behaviors, and mistreatment in GME environments^12–18,40^. Our findings extend this work by showing that discriminatory encounters remain prevalent even after IMGs and PRMGs attain full professional status. Language-based discrimination, often tied to accent, also remained common; however, distress associated with such discrimination decreased significantly from training to practice. This shift may reflect increased confidence, improved communication skills, or greater institutional stability later in physicians’ careers. Nevertheless, the persistence of discrimination across career stages underscores the need for systemic and longitudinal interventions^41–44^.

Another prominent finding was the considerable pressure to assimilate, manifested through changes in communication style, expression, and cultural practices. Participants frequently described modifying their accents, avoiding idiomatic expressions, or altering their appearance to conform to U.S. professional expectations. These pressures align with prior literature on acculturation challenges and cultural adaptation among IMGs, who often face expectations to perform in a manner consistent with U.S. norms while simultaneously experiencing identity dissonance^11,20,45,46^. Such pressures were closely linked to only moderate personal satisfaction in our study, suggesting a tension between cultural identity and professional integration. Research on bicultural and acculturation stress supports this dynamic, noting that individuals navigating multiple cultural expectations may experience emotional burden despite outward professional success^23,24^.

Despite these challenges, our participants reported high levels of professional satisfaction, largely driven by opportunities for growth, financial stability, and meaningful contributions to patient care. Many IMGs and PRMGs serve in underserved communities, care for diverse patient populations, and provide essential linguistic and cultural concordance, all of which have been associated with improved patient outcomes and satisfaction^28–33,38,47^. Our findings reinforce the essential role that IMGs and PRMGs play in the U.S. healthcare system, consistent with evidence showing their disproportionate representation in primary care, geriatrics, nephrology, rheumatology, and other high-need specialties^28,32,33^. This workforce contribution is particularly critical given persistent physician shortages and the growing racial, ethnic, and linguistic diversity of the U.S. population^31,37,39^.

The policy and institutional implications of our findings are substantial. Eliminating discrimination requires coordinated, institution-wide strategies, including transparent reporting structures, zero-tolerance policies, implicit-bias training, and culturally sensitive mentorship^43^Programs designed to promote belonging, such as peer support networks, wellness initiatives, and cultural appreciation efforts, may address the isolation and assimilation pressures described by participants^44,48–51^. Policies addressing visa constraints, immigration costs, and inequities in residency selection processes are also essential, given prior evidence demonstrating the unique burdens faced by IMGs and PRMGs, including financial strain and limited pathways to career advancement^16,19,49^. As the U.S. relies heavily on IMGs and PRMGs to maintain physician supply, particularly in underserved areas, building environments that support their well-being is essential to both workforce sustainability and health equity^28,29,36,52^.

This study has several limitations. Because the survey was shared through email and social media networks, we could not calculate a true response rate and may have oversampled younger physicians or those active on social media. Although the survey was available only in English, the majority of participants were undergoing or had completed U.S. medical training, where English proficiency is required. Additionally, as with all self-reported surveys, recall bias and social desirability bias remain possible. Nonetheless, the study includes a large, diverse, and geographically distributed group of IMGs and PRMGs across training levels and practice settings, enhancing the relevance of our findings.

In conclusion, our findings reveal a nuanced portrait of IMG and PRMG physicians in the U.S., one characterized by high professional satisfaction and meaningful career opportunities, yet also by persistent discrimination, pressures to assimilate, and only moderate personal fulfillment. Addressing these challenges is essential for fostering an equitable and sustainable physician workforce. Future research should examine specialty-specific experiences, evaluate interventions aimed at reducing discrimination and acculturation stress, and explore intersectional identities, including gender, race/ethnicity, and visa status, to better understand the long-term well-being and career trajectories of IMGs and PRMGs^53,54^.

## Supplementary Information

Below is the link to the electronic supplementary material.

ESM 1 (DOCX 50.6 KB)

## Data Availability

The full dataset for the study is not publicly available, but individuals interested in obtaining access to the full dataset can email. The study team has published the entire survey used for analysis to complement this.
