# Supplementary material for: Experiences of International and Puerto Rican Medical Graduates in the United States: A Cross-Sectional Survey
Source: J Gen Intern Med. 2026 Apr 14;41(10):2762–71. doi: 10.1007/s11606-026-10392-9 (PMC13421728; doi:10.1007/s11606-026-10392-9)
Supplement: Supplementary file 1 — (DOCX 50.6 KB) [file 11606_2026_10392_MOESM1_ESM.docx]

Appendix Table **1. Country of birth by region among IMGs and PRMGs (N=352)**

| **Region** | **Countries included*** | **n (%)** |
| --- | --- | --- |
| Latin America & Caribbean | Antigua, Argentina, Bolivia, Brazil, Chile, Colombia, Cuba, Dominican Republic, Ecuador, El Salvador, Guatemala, Honduras, Jamaica, Mexico, Nicaragua, Panama, Peru, Trinidad and Tobago, Uruguay, Venezuela, U.S. Puerto Rico, U.S. Virgin Islands | 171 (48.6) |
| South Asia | Bangladesh, India, Nepal, Pakistan, Sri Lanka, Kashmir, Myanmar | 61 (17.3) |
| Middle East / North Africa (MENA) | Egypt, Iraq, Israel, Jordan, Kuwait, Lebanon, Saudi Arabia, Sudan, Turkey, United Arab Emirates | 35 (9.9) |
| Sub-Saharan Africa | Cameroon, Ethiopia, Ghana, Kenya, Nigeria | 11 (3.1) |
| East & Southeast Asia | China, Japan, Taiwan | 3 (0.9) |
| Europe & Eurasia | Armenia, Azerbaijan, France, Germany, Greece, Hungary, Ireland, Italy, Kazakhstan, Moldova, Romania, Russia, Ukraine, United Kingdom, “Europe” | 20 (5.7) |
| North America (US/Canada) | Canada, United States | 24 (6.8) |
| Unknown / Missing | — | 27 (7.7) |
| **Total** |  | **352 (100)** |

*Country names listed as captured in the survey.

Appendix Table 2: Details about the migration by region of birth

|  | **Total**  **(N=352)** | | | | | | | |
| --- | --- | --- | --- | --- | --- | --- | --- | --- |
|  | **Latin America and the Caribbean** | **North America** | **South Asia** | **Middle East and North Africa (MENA)** | **Europe and Eurasia** | **Sub-Saharan Africa** | **East and Southeast Asia** | **Unknown** |
|  | **n=124** | **n=71** | **n=61** | **n=35** | **n=20** | **n=11** | **n=3** | **n=27** |
| **Reason for the migration*** | | | | | | | | |
| Health reasons | 1 (0.8) | 0 | 0 | 0 | 0 | 0 | 0 | 0 |
| Seeking refugee | 1 (0.8) | 0 | 0 | 1 (2.9) | 0 | 0 | 0 | 2 (7.4) |
| Leaving gang wars | 8 (6.5) | 1 (1.4) | 0 | 2 (5.7) | 0 | 0 | 0 | 0 |
| Avoiding civil wars | 4 (3.2) | 1 (1.4) | 2 (3.3) | 6 (17.1) | 0 | 2 (18.2) | 0 | 1 (3.7) |
| Escaping political oppression | 17 (13.7) | 1 (1.4) | 7 (11.5) | 9 (25.7) | 1 (5.0) | 2 (18.2) | 0 | 2 (7.4) |
| Looking for opportunities for family | 15 (12.1) | 11 (15.5) | 10 (16.4) | 13 (37.1) | 4 (20.0) | 4 (36.4) | 0 | 7 (25.9) |
| Family reasons | 15 (12.1) | 11 (15.5) | 18 (29.5) | 11 (31.4) | 9 (45.0) | 4 (36.4) | 0 | 4 (14.8) |
| Wanting professional recognition | 20 (16.1) | 12 (16.9) | 16 (26.2) | 15 (42.9) | 4 (20.0) | 0 | 1 (33.3) | 4 (14.8) |
| Pursuing Financial gains | 28 (22.6) | 18 (25.4) | 9 (14.8) | 8 (22.9) | 3 (15.0) | 3 (27.3) | 0 | 4 (14.8) |
| Lack of opportunities in home country | 66 (53.2) | 23 (32.4) | 22 (36.1) | 21 (60.0) | 6 (30.0) | 3 (27.3) | 0 | 11 (40.7) |
| Seeking professional gains | 70 (56.5) | 39 (54.9) | 29 (47.5) | 23 (65.7) | 8 (40.0) | 6 (54.5) | 3 (100.0) | 15 (55.6) |
| Looking for better Education | 89 (71.8) | 31 (43.7) | 31 (50.8) | 27 (77.1) | 7 (35.0) | 5 (45.5) | 2 (66.7) | 16 (59.3) |
| **Timing of the migration** | | | | | | | | |
| Elementary school | 1 (0.8) | 1 (1.4) | 0 | 0 | 0 | 0 | 0 | 0 |
| High school | 1 (0.8) | 1 (1.4) | 1 (1.6) | 0 | 0 | 0 | 0 | 0 |
| College | 1 (0.8) | 1 (1.4) | 0 | 1 (2.9) | 1 (5.0) | 0 | 0 | 2 (7.4) |
| After completing medical school | 93 (75.0) | 38 (53.5) | 51 (83.6) | 27 (77.1) | 9 (45.0) | 9 (81.8) | 2 (66.7) | 18 (66.7) |
| After completing residency | 12 (9.7) | 19 (26.8) | 6 (9.8) | 5 (14.3) | 5 (25.0) | 1 (9.1) | 0 | 4 (14.8) |
| After completing fellowship | 10 (8.1) | 5 (7.0) | 0 | 0 | 4 (20.0) | 0 | 1 (33.3) | 1 (3.7) |
| None of the above | 6 (4.8) | 6 (8.5) | 3 (4.9) | 2 (5.7) | 1 (5.0) | 1 (9.1) | 0 | 2 (7.4) |

*Multiple choice question

Appendix Table 3: Decision factor of going back home country and staying in the U.S. by region of birth

|  | **Total**  **(N=352)** | | | | | | | |
| --- | --- | --- | --- | --- | --- | --- | --- | --- |
|  | **Latin America and the Caribbean** | **North America** | **South Asia** | **Middle East and North Africa (MENA)** | **Europe and Eurasia** | **Sub-Saharan Africa** | **East and Southeast Asia** | **Unknown** |
|  | **n=124** | **n=71** | **n=61** | **n=35** | **n=20** | **n=11** | **n=3** | **n=27** |
| **Decision factor of going back home country*** | | | | | | | | |
| Toxic work environment | 6 (4.8) | 2 (2.8) | 1 (1.6) | 0 | 1 (5.0) | 1 (9.1) | 0 | 1 (3.7) |
| Family support | 3 (2.4) | 5 (7.0) | 3 (4.9) | 2 (5.7) | 0 | 0 | 0 | 1 (3.7) |
| Language | 11 (8.9) | 5 (7.0) | 2 (3.3) | 2 (5.7) | 3 (15.0) | 1 (9.1) | 0 | 4 (14.8) |
| Leadership opportunity | 19 (15.3) | 3 (4.2) | 1 (1.6) | 1 (2.9) | 0 | 4 (36.4) | 0 | 0 |
| Partners preference | 7 (5.6) | 9 (12.7) | 4 (6.6) | 0 | 3 (15.0) | 5 (45.5) | 0 | 1 (3.7) |
| Visa/immigration status | 13 (10.5) | 1 (1.4) | 14 (23.0) | 7 (20.0) | 1 (5.0) | 0 | 0 | 2 (7.4) |
| Professional opportunity | 21 (16.9) | 11 (15.5) | 4 (6.6) | 1 (2.9) | 1 (5.0) | 3 (27.3) | 1 (33.3) | 0 |
| Cost of living | 27 (21.8) | 12 (16.9) | 3 (4.9) | 2 (5.7) | 4 (20.0) | 1 (9.1) | 0 | 3 (11.1) |
| Family illness | 17 (13.7) | 6 (8.5) | 12 (19.7) | 10 (28.6) | 6 (30.0) | 0 | 1 (33.3) | 3 (11.1) |
| Food | 19 (15.3) | 8 (11.3) | 14 (23.0) | 4 (11.4) | 3 (15.0) | 2 (18.2) | 1 (33.3) | 10 (37.0) |
| Desire to raise children in home country | 17 (13.7) | 26 (36.6) | 11 (18.0) | 7 (20.0) | 3 (15.0) | 1 (9.1) | 1 (33.3) | 8 (29.6) |
| Feelings of isolation/lack of community | 19 (15.3) | 17 (23.9) | 17 (27.9) | 10 (28.6) | 3 (15.0) | 3 (27.3) | 0 | 7 (25.9) |
| Quality of life | 37 (29.8) | 22 (31.0) | 8 (13.1) | 6 (17.1) | 5 (25.0) | 6 (54.5) | 1 (33.3) | 8 (29.6) |
| Culture | 32 (25.8) | 23 (32.4) | 19 (31.1) | 9 (25.7) | 5 (25.0) | 2 (18.2) | 1 (33.3) | 14 (51.9) |
| Family | 80 (64.5) | 55 (77.5) | 41 (67.2) | 24 (68.6) | 13 (65.0) | 5 (45.5) | 3 (100.0) | 16 (59.3) |
| **Decision factor of staying in the U.S.*** | | | | | | | | |
| Refugee status | 5 (4.0) | 0 | 0 | 2 (5.7) | 0 | 0 | 0 | 0 |
| Looking for a change | 9 (7.3) | 3 (4.2) | 3 (4.9) | 4 (11.4) | 2 (10.0) | 1 (9.1) | 1 (33.3) | 0 |
| Avoiding political situations | 26 (21.0) | 1 (1.4) | 7 (11.5) | 6 (17.1) | 4 (20.0) | 3 (27.3) | 0 | 6 (22.2) |
| Partner is not willing to move back home | 14 (11.3) | 17 (23.9) | 7 (11.5) | 5 (14.3) | 5 (25.0) | 3 (27.3) | 0 | 4 (14.8) |
| Safety from wars violence | 30 (24.2) | 7 (9.9) | 5 (8.2) | 6 (17.1) | 3 (15.0) | 2 (18.2) | 0 | 4 (14.8) |
| Prefer to raise my family here | 30 (24.2) | 6 (8.5) | 9 (14.8) | 11 (31.4) | 6 (30.0) | 3 (27.3) | 1 (33.3) | 3 (11.1) |
| Lack of training opportunities in home country | 32 (25.8) | 15 (21.1) | 15 (24.6) | 8 (22.9) | 4 (20.0) | 3 (27.3) | 1 (33.3) | 9 (33.3) |
| Inability to pursue the same professional career | 33 (26.6) | 9 (12.7) | 7 (11.5) | 8 (22.9) | 7 (35.0) | 3 (27.3) | 1 (33.3) | 5 (18.5) |
| Professional recognition | 31 (25.0) | 11 (15.5) | 25 (41.0) | 14 (40.0) | 7 (35.0) | 2 (18.2) | 3 (100.0) | 9 (33.3) |
| Greater pay at work | 40 (32.3) | 30 (42.3) | 23 (37.7) | 11 (31.4) | 6 (30.0) | 4 (36.4) | 2 (66.7) | 10 (37.0) |
| Ability to seek professional growth promotion opportunities | 43 (34.7) | 21 (29.6) | 30 (49.2) | 18 (51.4) | 5 (25.0) | 3 (27.3) | 2 (66.7) | 7 (25.9) |
| Financial gain | 47 (37.9) | 30 (42.3) | 26 (42.6) | 12 (34.3) | 10 (50.0) | 5 (45.5) | 3 (100.0) | 11 (40.7) |
| Better and more work opportunities | 58 (46.8) | 33 (46.5) | 27 (44.3) | 13 (37.1) | 7 (35.0) | 6 (54.5) | 2 (66.7) | 10 (37.0) |

*Multiple choice question

Appendix Table 4: Personal and professional satisfaction while living in the U.S. by region of birth

|  | **Total**  **(N=352)** | | | | | | | |
| --- | --- | --- | --- | --- | --- | --- | --- | --- |
|  | **Latin America and the Caribbean** | **North America** | **South Asia** | **Middle East and North Africa (MENA)** | **Europe and Eurasia** | **Sub-Saharan Africa** | **East and Southeast Asia** | **Unknown** |
|  | **n=124** | **n=71** | **n=61** | **n=35** | **n=20** | **n=11** | **n=3** | **n=27** |
| **Personal satisfaction while living in US** | | | | | | | | |
| Not satisfied at all | 1 (0.8) | 0 | 3 (5.3) | 2 (5.7) | 0 | 0 | 0 | 1 (3.8) |
| Slightly satisfied | 11 (9.2) | 6 (8.8) | 9 (15.8) | 5 (14.3) | 3 (15.0) | 0 | 0 | 8 (30.8) |
| Moderately satisfied | 61 (51.3) | 38 (55.9) | 34 (59.6) | 16 (45.7) | 13 (65.0) | 9 (90.0) | 3 (100) | 10 (38.5) |
| Extremely satisfied | 46 (38.7) | 24 (35.3) | 11 (19.3) | 12 (34.3) | 4 (20.0) | 1 (10.0) | 0 | 7 (26.9) |
| **Professional satisfaction while living in US** | | | | | | | | |
| Not satisfied at all | 3 (2.5) | 2 (2.9) | 0 | 3 (8.6) | 1 (5.0) | 0 | 0 | 1 (4.0) |
| Slightly satisfied | 6 (5.0) | 3 (4.3) | 2 (3.7) | 6 (17.1) | 2 (10.0) | 0 | 0 | 3 (12.0) |
| Moderately satisfied | 43 (36.1) | 33 (47.1) | 35 (64.8) | 9 (25.7) | 9 (45.0) | 4 (40.0) | 1 (33.3) | 13 (52.0) |
| Extremely satisfied | 67 (56.3) | 32 (45.7) | 17 (31.5) | 17 (48.6) | 8 (40.0) | 6 (60.0) | 2 (66.7) | 8 (32.0) |

Appendix Table 5: Details about the migration by race, ethnicity, and medical graduate background

|  | **Total**  **(N=352)** | | **Total**  **(N=352)** | | **Total**  **(N=348)**** | |
| --- | --- | --- | --- | --- | --- | --- |
|  | **White** | **Non-White** | **Hispanic** | **Non-Hispanic** | **IMG** | **PRMG** |
|  | **n=158** | **n=194** | **n=180** | **n=172** | **n=289** | **n=59** |
| **Reason for the migration*** | | | | | | |
| Health reasons | 1 (0.6) | 0 | 1 (0.6) | 0 | 1 (0.3) | 0 |
| Seeking refugee | 3 (1.9) | 1 (0.5) | 2 (1.1) | 2 (1.2) | 3 (1.0) | 0 |
| Leaving gang wars | 8 (5.1) | 3 (1.5) | 9 (5.0) | 2 (1.2) | 9 (3.1) | 2 (3.4) |
| Avoiding civil wars | 6 (3.8) | 10 (5.2) | 4 (2.2) | 12 (7.0) | 15 (5.2) | 0 |
| Escaping political oppression | 21 (13.3) | 18 (9.3) | 19 (10.6) | 20 (11.6) | 37 (12.8) | 1 (1.7) |
| Looking for opportunities for family | 24 (15.2) | 40 (20.6) | 28 (15.6) | 36 (20.9) | 55 (19.0) | 9 (15.3) |
| Family reasons | 29 (18.4) | 43 (22.2) | 27 (15.0) | 45 (26.2) | 62 (21.5) | 8 (13.6) |
| Wanting professional recognition | 32 (20.3) | 40 (20.6) | 27 (15.0) | 45 (26.2) | 65 (22.5) | 7 (11.9) |
| Pursuing Financial gains | 34 (21.5) | 39 (20.1) | 46 (25.6) | 27 (15.7) | 55 (19.0) | 17 (28.8) |
| Lack of opportunities in home country | 72 (45.6) | 80 (41.2) | 85 (47.2) | 67 (39.0) | 130 (45.0) | 21 (35.6) |
| Seeking professional gains | 83 (52.5) | 110 (56.7) | 108 (60.0) | 85 (49.4) | 158 (54.7) | 34 (57.6) |
| Looking for better Education | 93 (58.9) | 115 (59.3) | 110 (61.1) | 98 (57.0) | 182 (63.0) | 24 (40.7) |
| **Timing of the migration** | | | | | | |
| Elementary school | 1 (0.6) | 1 (0.5) | 0 | 2(1.2) | 1 (0.3) | 0 |
| High school | 1 (0.6) | 2 (1.0) | 2 (1.1) | 1 (0.6) | 2 (0.7) | 1 (1.7) |
| College | 4 (2.5) | 2 (1.0) | 3 (1.7) | 3 (1.7) | 4 (1.4) | 1 (1.7) |
| After completing medical school | 101 (63.9) | 146 (75.3) | 116 (64.4) | 131 (76.2) | 219 (75.8) | 28 (47.5) |
| After completing residency | 27 (17.1) | 25 (12.9) | 34 (18.9) | 18 (10.5) | 32 (11.1) | 20 (33.9) |
| After completing fellowship | 13 (8.2) | 8 (4.1) | 16 (8.9) | 5 (2.9) | 15 (5.2) | 6 (10.2) |
| None of the above | 11 (7.0) | 10 (5.2) | 9 (5.0) | 12 (7.0) | 16 (5.5) | 3 (5.1) |

*Multiple choice question

** Four participants who selected neither IMG nor PRMG were removed.

Appendix Table 6: Decision factor of going back home country and staying in the U.S. by race, ethnicity, and medical graduate background

|  | **Total**  **(N=352)** | | **Total**  **(N=352)** | | **Total**  **(N=348)**** | |
| --- | --- | --- | --- | --- | --- | --- |
|  | **White** | **Non-White** | **Hispanic** | **Non-Hispanic** | **IMG** | **PRMG** |
|  | **n=158** | **n=194** | **n=180** | **n=172** | **n=289** | **n=59** |
| **Decision factor of going back home country*** | | | | | | |
| Toxic work environment | 7 (4.4) | 5 (2.6) | 8 (4.4) | 4 (2.3) | 10 (3.5) | 2 (3.4) |
| Family support | 10 (6.3) | 4 (2.1) | 9 (5.0) | 5 (2.9) | 8 (2.8) | 6 (10.2) |
| Language | 10 (6.3) | 18 (9.3) | 17 (9.4) | 11 (6.4) | 22 (7.6) | 6 (10.2) |
| Leadership opportunity | 14 (8.9) | 14 (7.2) | 20 (11.1) | 8 (4.7) | 25 (8.7) | 3 (5.1) |
| Partners preference | 12 (7.6) | 17 (8.8) | 13 (7.2) | 16 (9.3) | 22 (7.6) | 7 (11.9) |
| Visa/immigration status | 15 (9.5) | 23 (11.9) | 13 (7.2) | 25 (14.5) | 38 (13.1) | 0 |
| Professional opportunity | 22 (13.9) | 20 (10.3) | 28 (15.6) | 14 (8.1) | 34 (11.8) | 8 (13.6) |
| Cost of living | 21 (13.3) | 31 (16.0) | 35 (19.4) | 17 (9.9) | 41 (14.2) | 10 (16.9) |
| Family illness | 21 (13.3) | 34 (17.5) | 22 (12.2) | 33 (19.2) | 49 (17.0) | 6 (10.2) |
| Food | 14 (8.9) | 47 (24.2) | 23 (12.8) | 38 (22.1) | 55 (19.0) | 6 (10.2) |
| Desire to raise children in home country | 34 (21.5) | 40 (20.6) | 42 (23.3) | 32 (18.6) | 47 (16.3) | 27 (45.8) |
| Feelings of isolation/lack of community | 24 (15.2) | 52 (26.8) | 34 (18.9) | 42 (24.4) | 65 (22.5) | 11 (18.6) |
| Quality of life | 37 (23.4) | 56 (28.9) | 54 (30.0) | 39 (22.7) | 71 (24.6) | 21 (35.6) |
| Culture | 37 (23.4) | 68 (35.1) | 46 (25.6) | 59 (34.3) | 87 (30.1) | 18 (30.5) |
| Family | 100 (63.3) | 137 (70.6) | 122 (67.8) | 115 (66.9) | 187 (64.7) | 49 (83.1) |
| **Decision factor of staying in the U.S.*** | | | | | | |
| Refugee status | 5 (3.2) | 2 (1.0) | 4 (2.2) | 3 (1.7) | 4 (1.4) | 1 (1.7) |
| Looking for a change | 12 (7.6) | 11 (5.7) | 11 (6.1) | 12 (7.0) | 19 (6.6) | 3 (5.1) |
| Avoiding political situations | 27 (17.1) | 26 (13.4) | 29 (16.1) | 24 (14.0) | 50 (17.3) | 1 (1.7) |
| Partner is not willing to move back home | 23 (14.6) | 32 (16.5) | 29 (16.1) | 26 (15.1) | 41 (14.2) | 13 (22.0) |
| Safety from wars violence | 28 (17.7) | 29 (14.9) | 36 (20.0) | 21 (12.2) | 50 (17.3) | 6 (10.2) |
| Prefer to raise my family here | 38 (24.1) | 31 (16.0) | 34 (18.9) | 35 (20.3) | 61 (21.1) | 6 (10.2) |
| Lack of training opportunities in home country | 35 (22.2) | 52 (26.8) | 43 (23.9) | 44 (25.6) | 74 (25.6) | 11 (18.6) |
| Inability to pursue the same professional career | 36 (22.8) | 37 (19.1) | 44 (24.4) | 29 (16.9) | 62 (21.5) | 10 (16.9) |
| Professional recognition | 41 (25.9) | 61 (31.4) | 37 (20.6) | 65 (37.8) | 96 (33.2) | 6 (10.2) |
| Greater pay at work | 49 (31.0) | 77 (39.7) | 67 (37.2) | 59 (34.3) | 97 (33.6) | 28 (47.5) |
| Ability to seek professional growth promotion opportunities | 44 (27.8) | 85 (43.8) | 60 (33.3) | 69 (40.1) | 110 (38.1) | 18 (30.5) |
| Financial gain | 60 (38.0) | 84 (43.3) | 72 (40.0) | 72 (41.9) | 117 (40.5) | 24 (40.7) |
| Better and more work opportunities | 65(41.1) | 91 (46.9) | 84 (46.7) | 72 (41.9) | 122 (42.2) | 31 (52.5) |

*Multiple choice question

** Four participants who selected neither IMG nor PRMG were removed.

Appendix Table 7: Personal and professional satisfaction while living in the U.S. by race, ethnicity, and medical graduate background

|  | **Total**  **(N=352)** | | **Total**  **(N=352)** | | **Total**  **(N=348)*** | |
| --- | --- | --- | --- | --- | --- | --- |
|  | **White** | **Non-White** | **Hispanic** | **Non-Hispanic** | **IMG** | **PRMG** |
|  | **n=158** | **n=194** | **n=180** | **n=172** | **n=289** | **n=59** |
| **Personal satisfaction while living in US** | | | | |  |  |
| Not satisfied at all | 3 (2.0) | 4 (2.2) | 0 | 7 (4.3) | 7 (2.5) | 0 |
| Slightly satisfied | 11 (7.2) | 31 (16.8) | 19 (10.8) | 23 (14.2) | 34 (12.2) | 8 (14.0) |
| Moderately satisfied | 70 (45.8) | 114 (61.6) | 85 (48.3) | 99 (61.1) | 155 (55.8) | 27 (47.4) |
| Extremely satisfied | 69 (45.1) | 36 (19.5) | 72 (40.9) | 33 (20.4) | 82 (29.5) | 22 (38.6) |
| **Professional satisfaction while living in US** | | | | |  |  |
| Not satisfied at all | 5 (3.3) | 5 (2.7) | 5 (2.9) | 5 (3.1) | 8 (2.9) | 2 (3.5) |
| Slightly satisfied | 8 (5.2) | 14 (7.7) | 8 (4.6) | 14 (8.7) | 19 (6.9) | 3 (5.3) |
| Moderately satisfied | 54 (35.3) | 93 (50.8) | 63 (36.0) | 84 (52.2) | 122 (44.2) | 22 (38.6) |
| Extremely satisfied | 86 (56.2) | 71 (38.8) | 99 (56.6) | 58 (36.0) | 127 (46.0) | 30 (52.6) |

* Four participants who selected neither IMG nor PRMG were removed.
